# Visualization of MMP-2 Activity Using Dual-Probe Nanoparticles to Detect Potential Metastatic Cancer Cells

**DOI:** 10.3390/nano8020119

**Published:** 2018-02-21

**Authors:** Aeju Lee, Sung Hoon Kim, Hyun Lee, Bohee Kim, Yoon Suk Kim, Jaehong Key

**Affiliations:** 1 International Research Organization for Advanced Science and Technology (IROAST), Kumamoto University, Kumamoto 860-8555, Japan; aeju-lee@kumamoto-u.ac.jp; 2 Magnesium Research Center, Kumamoto University, Kumamoto 860-8555, Japan; aeju-lee@kumamoto-u.ac.jp; 3 Department of Biomedical Laboratory Science, College of Health Sciences, Yonsei University, Wonju, Gangwon-do 26493, Korea; yoonsukkim@yonsei.ac.kr (B.K.); yoonsukkim@yonsei.ac.kr (Y.S.K.); 4 Department of Biomedical Engineering, Yonsei University, Wonju, Gangwon-do 26493, Korea; jkey@yonsei.ac.kr

**Keywords:** active matrix metalloproteinase-2, PLGA-PEI nanoparticles, imaging sensor, metastasis

## Abstract

Matrix metalloproteinases (MMPs) are a family of zinc-dependent enzymes capable of degrading extracellular matrix components. Previous studies have shown that the upregulation of MMP-2 is closely related to metastatic cancers. While Western blotting, zymography, and Enzyme-Linked Immunosorbent Assays (ELISA) can be used to measure the amount of MMP-2 activity, it is not possible to visualize the dynamic MMP-2 activities of cancer cells using these techniques. In this study, MMP-2-activated poly(lactic-co-glycolic acid) with polyethylenimine (MMP-2-PLGA-PEI) nanoparticles were developed to visualize time-dependent MMP-2 activities. The MMP-2-PLGA-PEI nanoparticles contain MMP-2-activated probes that were detectable via fluorescence microscopy only in the presence of MMP-2 activity, while the Rhodamine-based probes in the nanoparticles were used to continuously visualize the location of the nanoparticles. This approach allowed us to visualize MMP-2 activities in cancer cells and their microenvironment. Our results showed that the MMP-2-PLGA-PEI nanoparticles were able to distinguish between MMP-2-positive (HaCat) and MMP-2-negative (MCF-7) cells. While the MMP-2-PLGA-PEI nanoparticles gave fluorescent signals recovered by active recombinant MMP-2, there was no signal recovery in the presence of an MMP-2 inhibitor. In conclusion, MMP-2-PLGA-PEI nanoparticles are an effective tool to visualize dynamic MMP-2 activities of potential metastatic cancer cells.

## 1. Introduction

Cancer is one of the major diseases affecting humans throughout the world. Over the past decades, many research groups have studied cancer cell mutations related to proliferation, survival, and metastasis. Recently, it has been recognized that the tumor microenvironment (TME), in addition to fibroblasts and the extracellular matrix (ECM), is an important factor influencing tumor progression [1]. It has been shown that constant interactions between the tumor and TME influence the growth and metastasis of cancer [2].

Matrix metalloproteinases (MMPs) are a family of zinc-dependent enzymes that are capable of degrading ECM components. They are present as pro-MMP forms in healthy individuals and play a positive role in physiological processes such as wound healing and pregnancy [3,4]. In a healthy state, a homeostatic balance between MMPs and tissue inhibitors of MMPs is maintained [5]. However, dysregulation of this balance can contribute to the malignancy of the tumor [6]. Several studies have suggested that many cancers show an upregulation of MMPs, and this upregulation is closely related to a worse prognosis and aggressive tumor behavior [7,8,9,10,11]. Among the different MMPs, MMP-2 (gelatinase A) is known as one of the major contributors to metastasis and angiogenesis. Itoh et al. demonstrated that MMP-2 plays an important role in angiogenesis and tumor progression in mice intradermally implanted by B16-BL6 melanoma cells or Lewis lung carcinoma cells [7]. Xu et al. showed that MMP-2 contributes to cell migration by interacting with collagen, excluding the potential overlapping effects of MMP-9 using MMP-9^−^/^−^ cells [10]. Dong et al. found that the expression of MMP-2 was much higher in colorectal cancer than in normal colorectal tissues, which indicated that high levels of MMP-2 were linked with the tumor size, lymph node metastasis, and tumor invasion [11]. Munoz-Najar also indicated that MMP-2 activation was overexpressed under hypoxic microenvironments and stimulated breast carcinoma cell invasion [12]. Besides this, many human tumors including glioblastomas, melanomas, breast, and colon cancer show upregulated MMP-2 activity [13]. Zheng et al. have demonstrated that MMP-2 plays a leading role in the angiogenesis of gastric carcinomas [14]. Thus, these studies suggest that overexpressed MMP-2 is a potential marker of metastasis.

Several methods such as Western blotting, zymography, and Enzyme-Linked Immunosorbent Assay (ELISA) have been applied for the detection of active MMP-2 in biological samples [15,16]. However, all these methods suffer from limitations in the detection of dynamic MMP-2 activities and visualization of the distribution of MMP-2 activity in cancer cells and their microenvironment. Although Western blot analysis is commonly used for protein analysis, the samples are prepared by either homogenizing the tumor or isolating it via total cell lysates. Gel zymography is an electrophoretic technique that is one of the most commonly utilized methods for monitoring MMPs [17]. However, it is a time-consuming process that involves the staining and destaining of acrylamide gels, which can lead to a loss of sensitivity or bleaching of the bands that will make them ineligible for quantification purposes. ELISA is an assay for the quantitative measurement of pro-MMPs and active MMPs, but it also requires cell culture supernatant or cell lysates. Thus, all these methods are limited in tracking active MMPs from intact cells or tissues.

Previously, we reported the preparation and application of an MMP-2-activated fluorogenic peptide sensor (MMP-2 probe) [18]. In this study, we prepared MMP-2-activated poly(lactic-co-glycolic acid) with polyethylenimine (MMP-2-PLGA-PEI) nanoparticles and further investigated whether they could visualize an active MMP-2 signal, differentiating between the MMP-2-positive and -negative cells. Herein, the MCF-7 and HaCat cell lines were selected as MMP-2-negative and MMP-2-positive, respectively, which was confirmed by the reverse transcription polymerase chain reaction (RT-PCR) analysis of MMP-2 mRNA levels from various human cancer cell lines including A549, MCF-7, HeLa, AGS, HaCat, HCT15, and 293A (Supplementary Figure S1). The MMP-2-PLGA-PEI nanoparticles were composed of dual probes: the first probe was used to visualize MMP-2 activity, and the second one continuously indicated the location of the nanoparticles in an in vitro system. The MMP-2-PLGA-PEI nanoparticles were validated by recombinant active enzyme and confocal imaging of MMP-2-positive or -negative cell lines. Our results suggest that the MMP-2-PLGA-PEI nanoparticles are a promising tool for visualizing the dynamic MMP-2 activities of potential metastatic cancer cells.

## 2. Materials and Methods 

### 2.1. Chemicals

Poly(dl-lactide-co-glycolic acid) (PLGA, lactide/glycolide 50:50, Molecular weight, *M*_w_ = 38,000–54,000) and polyethylenimine (PEI, branched, *M*_w_ = ~25,000) were purchased from Sigma-Aldrich (St. Louis, MO, USA). 1,2-Dioleoyl-sn-glycero-3-phosphoethanolamine-*N*-(lissamine rhodamine B sulfonyl) (RhoB lipid) was acquired from Avanti Polar Lipids, Inc. (Alabaster, AL, USA). Cyanine 5 (Cy5) near-infrared (NIR) fluorescence dye was procured from Lumiprobe (Hannover, Germany), and Black Hole Quencher-3 (BHQ-3) was purchased from Bioresearch Technologies (Petaluma, CA, USA). Matrix metalloproteinase-2 peptide substrate was a customized product from Peptron (Daejeon, Korea).

### 2.2. Synthesis and Characterization of MMP-2-Activated Peptide Sensor

The MMP-2-activated peptide sensor was synthesized as previously reported [16,18]. Briefly, NIR fluorescence dye (Cy5, Ex/Em: 646/662) and BHQ-3 (absorbance: 650 nm) were conjugated to the MMP-2 peptide substrate Gly-Pro-Leu-Gly-Val-Arg-Gly-Lys-Gly-Gly (the cleavage site is indicated by italics). The Cy5 succinimide ester attached to the N-terminal glycine was conjugated and purified by reversed-phase high-performance liquid chromatography (RP-HPLC). The tert-butyloxycarbonyl (Boc)-protected primary amine of lysine was deprotected by Trifluoroacetic acid (TFA) and reacted with BHQ-3 succinimide ester overnight. To confirm the MMP-2-activated sensor specificity, 50 nM solutions of each of the activated recombinant enzymes (MMP-1, -2, -3, -7, -9, and -13) were prepared and reacted with 30 nM solutions of the MMP-2-activated sensor in TCNB (50 mM Tris with 10 mM CaCl_2_, 150 mM NaCl, and 0.05% Brij 35; pH 7.5) reaction buffer under light protection. The fluorescence signal was detected every 10 min at 37 °C. The fluorescence recovery of the MMP-2-activated peptide sensor was examined by HPLC and fluorescence sodium dodecyl sulfate polyacrylamide gel electrophoresis (SDS-PAGE) after incubation with recombinant MMP-2 for 30 min.

### 2.3. Preparation of PLGA-PEI Nanoparticles

PLGA-PEI nanoparticles were synthesized via a nanoprecipitation method [19]. Briefly, PLGA (6.5 mg) was dissolved in 650 μL of acetonitrile. RhoB lipid (6.5 μL) was added and thoroughly mixed by repeated pipetting. PEI (6.3 mg) was dissolved in 65 μL of deionized water and gently vortexed. 1-Ethyl-3-(3-dimethylaminopropyl) carbodiimide hydrochloride (EDC, 65 mg) and *N*-hydroxysuccinimide (NHS, 65 mg) were dissolved in 1.3 mL of phosphate-buffered saline (PBS, pH 7.4). The EDC/NHS solution was added to PEI in a dropwise manner, followed by the addition of deionized water to achieve a volume of 6.5 mL. The reaction was performed for 20 min at room temperature. After 20 min, PLGA containing RhoB lipid was added to the PEI solution in a dropwise manner and the reaction mixture was stirred overnight at 400 rpm at room temperature under dark conditions. To remove the unconjugated ingredients, the solution was dialyzed against deionized water for a day (molecular weight cut-off, (Molecular weight cut-off) MWCO = 10 kDa).

### 2.4. Conjugation of MMP-2-Activated Peptide Sensor with PLGA-PEI Nanoparticles

To conjugate the MMP-2-activated peptide sensor with the PLGA-PEI nanoparticles to produce MMP-2-PLGA-PEI nanoparticles, 3.5 mg of the MMP-2-activated peptide sensor was dissolved in 200 μL of dimethyl sulfoxide (DMSO)/PBS (1:1, *v*/*v*). Then, EDC (32.5 mg) and NHS (32.5 mg) were dissolved in 100 μL of PBS and added to the solution of the MMP-2-activated peptide sensor in a dropwise manner. After reacting for 20 min at room temperature, the sensor mixture was added to the PLGA-PEI nanoparticle solution and incubated for 12 h at room temperature under dark conditions. Dialysis against deionized water was performed for a day (MWCO = 10 kDa). The final product was centrifuged (13,500 rpm, 15 min), and the MMP-2-PLGA-PEI nanoparticles in the pellet were lyophilized.

### 2.5. Size, Stability, and Morphology Characterization of MMP-2-PLGA-PEI Nanoparticles

The size and zeta potential of the MMP-2-PLGA-PEI nanoparticles were confirmed by a Zetasizer Nano (Malvern Instruments, Worcestershire, UK). For size measurements, the nanoparticles were resuspended in water or PBS. The stability of each group was confirmed under the same conditions at 37 °C for up to 7 days. For scanning electron microscopy (SEM) images, a drop of the samples was spotted on a silicon template and dried at room temperature. The dried samples were sputtered with platinum to increase the contrast and signal-to-noise ratio. For transmission electron microscopy (TEM) images, a drop of the sample was spotted on a grid and stained with 0.01% tungsten solution for 5 min. The sample was dried in a desiccator.

### 2.6. NIR Fluorescence Signal Recovery of MMP-2-PLGA-PEI Nanoparticles by Active Recombinant Enzyme

To confirm the NIR fluorescence signal recovery, 10 nM of the MMP-2-activated peptide sensor or MMP-2-PLGA-PEI nanoparticles were incubated with active recombinant MMP-2 (50 nM) with or without the inhibitor (10 nM). The fluorescence signal was detected every 10 min at 37 °C. TCNB buffer was used as a control.

### 2.7. NIR Fluorescence Signal Recovery of MMP-2-PLGA-PEI Nanoparticles by Culture Media and Cell Lysate

Cell culture media and cell lysates from HaCat (MMP-2-positive) and MCF-7 (MMP-2-negative) cells were used to confirm the fluorescence recovery of the MMP-2-PLGA-PEI nanoparticles. The cells were washed with PBS and then lysed at 4 °C with a lysis buffer containing 1% Triton X-100, protease inhibitor cocktail, phosphatase inhibitor cocktail, and PBS. The lysates were centrifuged and the collected supernatants were used as the cell lysate. MMP-2-PLGA-PEI nanoparticles (10 nM) were incubated with 400 μL of either the culture media or cell lysate, and the fluorescence signals were detected every 10 min at 37 °C. Fresh media and lysis buffer were used as controls.

### 2.8. In Vitro Cytotoxicity of MMP-2-PLGA-PEI Nanoparticles

MCF-7 and HaCat cells were seeded into 6-well plates at 2 × 10^5^ cells/well in complete media and incubated for 24 h. The cell media was changed to serum-free media and the cells were incubated for a further 24 h. Subsequently, the cells were treated with five different concentrations (0 to 160 μg/mL) of nanoparticles for 30 min and then detached using 0.25% trypsin. Next, they were washed and stained with trypan blue. Nonstained viable cells were counted using a hemocytometer.

### 2.9. Confocal Microscopy

Cells (4 × 10^4^/well) were seeded in 24-well plates in complete media and cultured for 24 h, followed by a further incubation in serum-free media. After incubation for 3 h and 24 h, MMP-2-PLGA-PEI nanoparticles (80 μg/mL) were added to the plates for 30 min. The cells were washed twice with PBS, fixed in 4% paraformaldehyde for 15 min at room temperature, and washed again twice with PBS. After addition of the mounting medium containing 4’,6-diamidino-2-phenylindole (DAPI), the cells were imaged using a Zeiss LSM 710 Confocal laser scanning microscope (Carl Zeiss).

### 2.10. RNA Isolation and RT-PCR

Total RNA was extracted using Trizol^®^ reagent according to the manufacturer’s instructions (Invitrogen, Waltham, MA, USA). cDNAs were synthesized by reverse transcription with 2 μg of total RNA, 0.25 μg of a random hexamer, and 200 units of Maloney murine leukemia virus reverse transcriptase (MMLV-RT) for 10 min at 25 °C, 50 min at 37 °C, and 15 min at 70 °C. The cDNAs were amplified by PCR using Prime *Taq* premix PCR kit (Genet Bio, Chungnam, Korea). The primer sequences for MMP-2 were forward 5’-TTTCCATTCCGCTTCCAGGGCAC-3’ and reverse 5’-TCGCACACCACATCTTTCCGTCACT-3’, and thermal cycling conditions were as follows: 5 min at 94 °C (initial denaturation); 25 cycles of 30 s at 94 °C (denaturation), 30 s at 65 °C (annealing), and 30 s at 72 °C (extension); and 7 min at 72 °C (final extension). Primer sequences for glyceraldehyde 3-phosphate dehydrogenase (GAPDH) were forward 5’-CGGGAAGCTTGT CATCAATGG-3’ and reverse 5’GGCAGTGATGGCATGGACTG-3’, and cycling conditions were 5 min at 94 °C (initial denaturation); 18 cycles of 30 s at 94 °C (denaturation), 30 s at 55 °C (annealing), and 30 s at 72 °C (extension); and 7 min at 72 °C (final extension). The PCR products were confirmed by electrophoresis on 2% (*w*/*v*) agarose gels containing 0.5 μg/mL ethidium bromide. The product size was determined using a 100-bp DNA ladder. Gel images were taken using the Gel Doc^TM^ XR+ system (Bio-Rad, Hercules, CA, USA).

### 2.11. Statistical Analysis

Data are presented as mean ± SD. Statistical differences were determined by two-way Analysis of Variance, ANOVA (*n* = 3).

## 3. Results and Discussion

### 3.1. Characterization of the MMP-2-Activated Peptide Sensor

**Figure 1:**
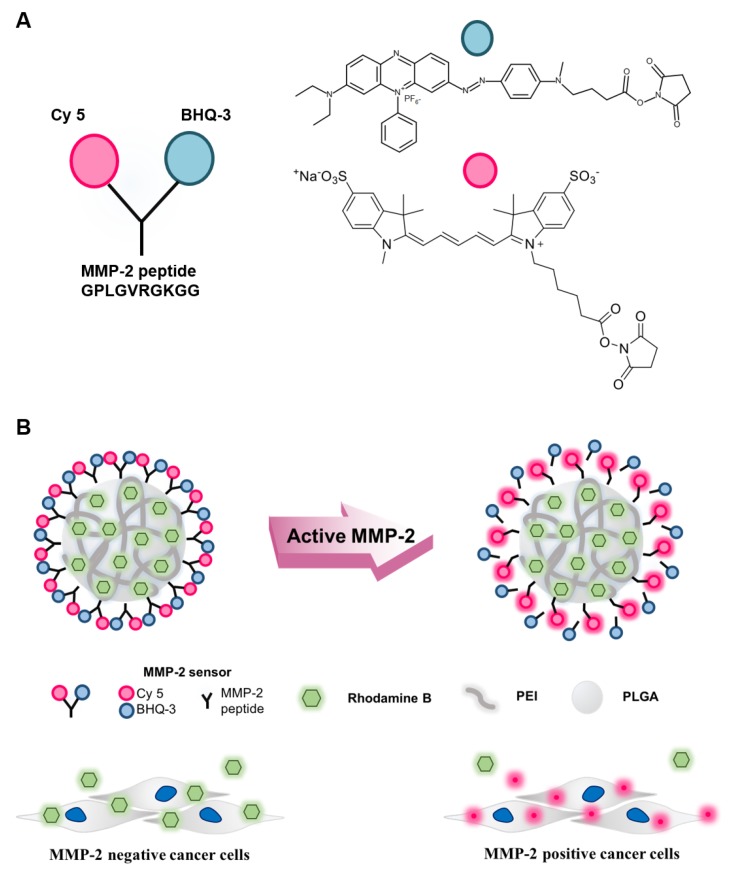
Schematic illustration of the matrix metalloproteinase-2 (MMP-2)-activated peptide sensor and MMP-2-activated poly(lactic-co-glycolic acid) with polyethylenimine (MMP-2-PLGA-PEI) nanoparticles. (**A**) Chemical structure of the MMP-2-activated peptide sensor. The sensor consisted of a near-infrared fluorescence dye (Cy5), MMP-2 substrate peptide, and a dark quencher (BHQ-3); (**B**) Schematic diagram of the MMP-2-PLGA-PEI nanoparticles. The fluorescence signal was recovered only in the presence of MMP-2-positive cancer cells.

**Figure 2:**
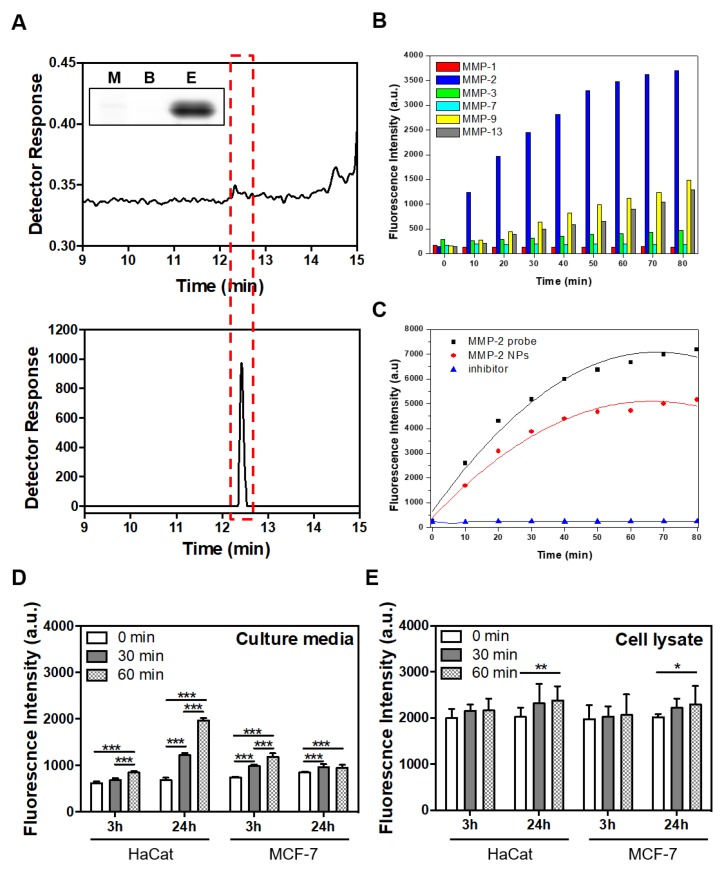
In vitro characterization of the MMP-2-activated peptide sensor and MMP-2-PLGA-PEI nanoparticles. (**A**) Analysis of MMP-2-activated peptide sensor fluorescence recovery by SDS-PAGE (upper panel; inset box: M-marker, B-TCNB buffer, E-Enzyme) and HPLC analysis results with MMP-2 enzyme (lower panel) and without MMP-2 enzyme (upper panel) indicated by the red dotted box. The MMP-2-activated peptide sensor was incubated with active recombinant MMP-2 for 30 min; (**B**) Signal recovery after incubation of the MMP-2-activated peptide sensor in the presence of various recombinant MMPs (MMP-1, -2, -3, -7, -9, -13) for 80 min at 37 °C; (**C**) MMP-2 fluorescence signal recovery from the MMP-2-activated peptide sensor, MMP-2-PLGA-PEI nanoparticles, and inhibitor-treated MMP-2-activated peptide sensor during incubation with recombinant MMP-2 for 80 min at 37 °C; (**D**) Analysis of MMP-2-PLGA-PEI fluorescence recovery after incubation with cell culture media; (**E**) MMP-2-PLGA-PEI fluorescence recovery of the cell lysate from MCF-7 and HaCat cells. Cell culture media and cell lysates were collected after culturing in fresh media for 3 h and 24 h. MMP-2 fluorescence signal recovery was confirmed at 0, 30, and 60 min. (* indicates *p* < 0.05, ** indicates *p* < 0.01, *** indicates *p* < 0.005).

The MMP-2-activated peptide sensor consisted of Cy5, BHQ-3, and MMP-2-specific peptide (Cy5-GPLGVRGK(BHQ-3)GG, purity: >98%) (Figure 1A). The MMP-2-activated peptide sensor was incubated with TCNB buffer or activated human recombinant MMP-2 enzyme. Clear MMP-2 signal recovery was detected by HPLC at a 12.3 min retention time in the presence of MMP-2 (Figure 2A). Significant MMP-2 recovery was also detected on SDS-PAGE gel (Figure 2A, inset). The enzyme specificity of the MMP-2-activated peptide sensor was confirmed by incubation with active recombinant MMP-1, -2, -3, -7, -9, or -13. The NIR fluorescence signal demonstrated time-dependent recovery with MMP-2, MMP-9, and MMP-13 (18.8-fold, 7.2-fold, and 6.9-fold, respectively). However, there was no recovery of NIR fluorescence signals against MMP-1, -3, and -7 (Figure 2B).

### 3.2. Characterization of the MMP-2-PLGA-PEI Nanoparticles

The MMP-2-PLGA-PEI nanoparticles were composed of PLGA-PEI, which entrapped the RhoB lipid. The MMP-2-activated peptide sensor was conjugated onto the surface of the PLGA-PEI nanoparticles. The nanoparticles successfully recovered fluorescence signals from MMP-2-positive cells (Figure 1B). Time-dependent recovery of fluorescent signals of the MMP-2-activated peptide sensor and MMP-2-PLGA-PEI nanoparticles was confirmed by incubation with active recombinant MMP-2 with or without the MMP-2 inhibitor. The NIR fluorescence signals demonstrated that the MMP-2-activated peptide sensor and MMP-2-PLGA-PEI nanoparticles recovered the fluorescence signal with 19.1- and 15.6-fold enhancement, respectively. However, there was no recovery in the presence of an MMP-2 inhibitor (Figure 2C). To confirm the fluorescence signal recovery of MMP-2-PLGA-PEI nanoparticles at a cellular level, the sensors were incubated with cell culture media and cell lysates. Cell culture media from both HaCat and MCF-7 cells were collected after 3 h and 24 h incubation. The culture media was incubated again with the MMP-2-PLGA-PEI nanoparticles for 0, 30, and 60 min, and the recovered fluorescence intensities were measured. When the results of 0 min and 60 min incubation experiments were compared, it was found that the culture media from HaCat cells showed 1.4- and 3.18-fold increased MMP-2 fluorescence intensity at 3 h and 24 h, respectively. The culture media from MCF-7 cells showed 1.02- and 1.22-fold increased MMP-2 fluorescence intensity at 3 h and 24 h, respectively (Figure 2D). This result clearly demonstrated that the MMP-2-PLGA-PEI nanoparticles could detect the active MMP-2 enzymes secreted from these cells, differentiating HaCat from MCF-7. HaCat and MCF-7 cell lysates were prepared using the lysis buffer described above. It was evident from a comparison of the results of the 0 min and 60 min incubation experiments that the HaCat and MCF-7 cell lysates showed 1.17-fold and 1.14-fold increased MMP-2 fluorescence intensities, respectively. This result suggested that there was a substantially smaller difference in the MMP-2 signals between these cells. This was because most active MMP-2 enzymes are secreted outside of the cells and do not stay inside the cells [13,20]. Interestingly, the MMP-2-PLGA-PEI nanoparticles detected higher MMP-2 enzyme activities inside the HaCat cells (*p* < 0.01) than the MMP-2 activity inside the MCF-7 cells (*p* < 0.05) (Figure 2E).

The expression of MMP-2 is closely related to metastasis and prognosis [21,22]. In tumor angiogenesis, MMP-2 and MMP-9 have been shown to promote the “angiogenic switch” [23]. In general, MMP-9 is known to induce the release of vascular endothelial growth factor (VEGF) in the ECM. MMP-2 activity is necessary to activate pro-MMP-9 [24]. Therefore, the measurement of active MMP-2 could be a promising approach to early diagnosis of cancer metastasis. MMP-2 is secreted into the ECM as a proenzyme and gets activated. For this reason, active MMP-2 expression was low in the cell lysates of both cell lines (Figure 2E). In contrast, a clear increase in MMP-2 activity was observed for HaCat cell culture media. This is especially noteworthy considering that the concentration of active MMP-2 would be very low given the volume of culture media used (10 mL in a 100 mm dish) (Figure 2D). These results suggest that the MMP-2-PLGA-PEI nanoparticles can be a promising tool for an accurate and sensitive detection of active MMP-2 at a low concentration.

**Figure 3:**
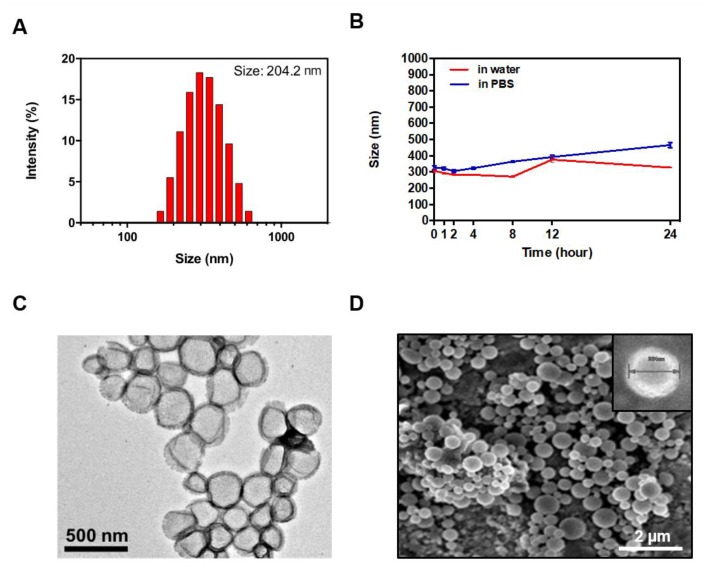
Characterization of MMP-2-PLGA-PEI nanoparticles. (**A**) Size distribution in aqueous solutions; (**B**) Stability tests at 37 °C in water and physiological solution (PBS, pH 7.4); (**C**) Transmission emission microscopy (TEM) images of the nanoparticles; (**D**) Scanning electron microscopy (SEM) images of the nanoparticles.

The MMP-2-PLGA-PEI nanoparticles showed a mono-dispersed size in the aqueous solution, as confirmed by dynamic light scattering (DLS). The MMP-2-PLGA-PEI nanoparticles presented a size of 204.2 ± 69.4 nm with zeta potential of 60.1 ± 7.73 mV (Figure 3A). To determine their stability, the MMP-2-PLGA-PEI nanoparticles were re-suspended in water or PBS. Both samples were incubated at 37 °C, and the hydrodynamic diameter of the nanoparticles was analyzed after 1, 2, 4, 8, 12, and 24 h by DLS (Figure 3B). The morphology of the MMP-2-PLGA-PEI nanoparticles was confirmed by TEM and SEM imaging. The TEM images showed the presence of PEI on the surface of the nanoparticles (Figure 3C). The SEM images confirmed the spherical shape of the nanoparticles in aqueous solutions.

### 3.3. Confirmation of MMP-2 Gene Expression, Cellular Cytotoxicity, and Cell Internalization

**Figure 4:**
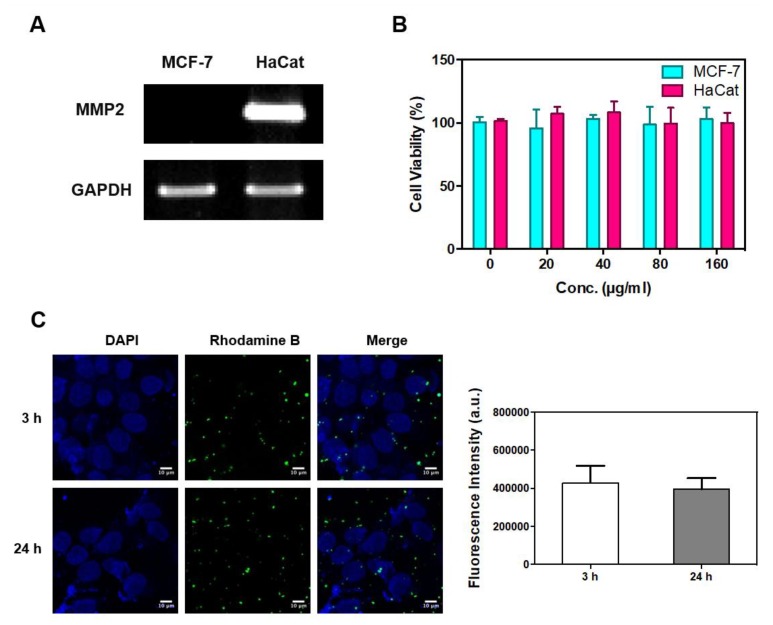
Confirmation of MMP-2 expression, cellular cytotoxicity, and MMP-2-PLGA-PEI nanoparticle uptake. (**A**) MMP-2 mRNA level in MCF-7 and HaCat cells was analyzed by RT-PCR. Glyceraldehyde 3-phosphate dehydrogenase (GAPDH) was used as an internal control; (**B**) In vitro cell cytotoxicity analysis for MCF-7 (cyan) and HaCat (pink) cells after a 30 min incubation with PLGA-PEI nanoparticles; (**C**) Confocal microscopy analysis of cellular uptake of Rhodamine B lipid PLGA-PEI nanoparticles. Results are presented as mean ± SD (*n* = 3) (Scale bar: 10 µm).

Based on previous research, MCF-7 and HaCat cells were selected as candidate MMP-2-negative and -positive cells, respectively [25,26]. A comparison of the MMP-2 mRNA expression of the two cell lines showed that the HaCat cells strongly expressed MMP-2 whereas the MCF-7 cells did not (Figure 4A). Next, the cytotoxicity of MMP-2-PLGA-PEI nanoparticles on MCF-7 and HaCat cells was investigated. Each cell line was treated with a different concentration of MMP-2-PLGA-PEI nanoparticles (0 to 160 μg/mL), and cell survival was assessed after incubation for 30 min. It was found that the MMP-2-PLGA-PEI nanoparticles were not toxic to MCF-7 and HaCat cells even at a concentration of 160 μg/mL, as shown in Figure 4B. Confocal microscopy using RhoB confirmed that there was no significant difference in the internalization of MMP-2-PLGA-PEI nanoparticles between 3 and 24 h incubation (Figure 4C). In this study, biodegradable and biocompatible PLGA nanoparticles were modified by a cationic agent (PEI) to enhance cell permeability [27]. As PEI has a strong positive charge in aqueous solutions, the MMP-2-PLGA-PEI nanoparticles were easily attached to the cell surface and induced cell internalization within 30 min with no significant cytotoxicity (Figure 4B,C). These results suggest that the MMP-2-PLGA-PEI nanoparticles can be efficiently applied not only for sensing MMP-2, but also for the delivery of therapeutic agents such as small interfering MMP-2 genes.

### 3.4. Evaluation of Cellular Active MMP-2 Using MMP-2-PLGA-PEI Nanoparticles

**Figure 5:**
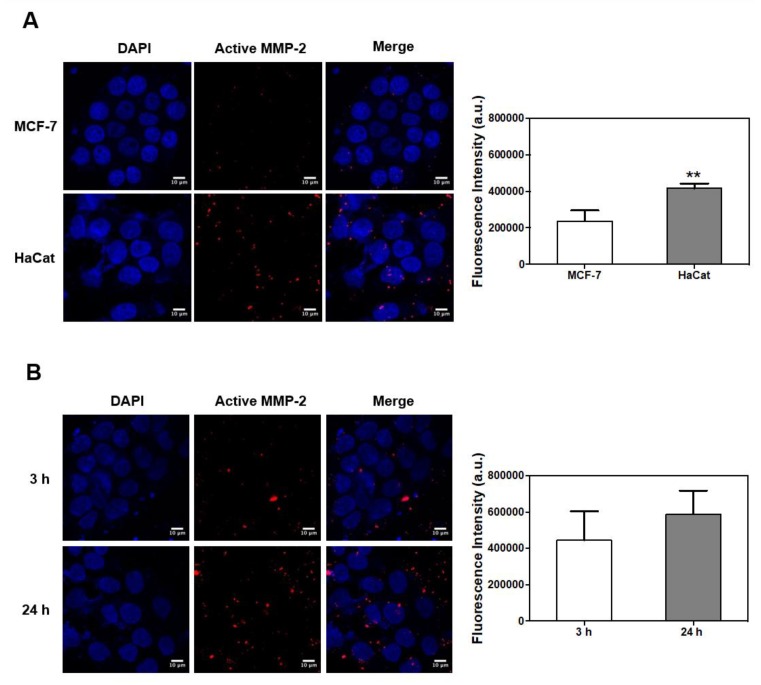
MMP-2 fluorescence signal recovery according to cell line and culture time. (**A**) Activity of MMP-2 in MCF-7 and HaCat cells by confocal imaging using MMP-2-PLGA-PEI nanoparticles. After culturing in fresh media for 24 h, the cells were incubated with MMP-3-PLGA-PEI nanoparticles for 30 min. Mean signal intensities were calculated by the imaging software (*n* = 3); (**B**) Cell culture time–dependent activity of MMP-2 in HaCat cells. Cells were precultured in serum-free media for 3 h and 24 h, respectively, and then incubated with MMP-2-PLGA-PEI nanoparticles for 30 min. Mean signal intensities were calculated by the imaging software (*n* = 3). (** indicates *p* < 0.01.) (Scale bar: 10 µm).

To confirm the MMP-2-specific recovery of MMP-2-PLGA-PEI nanoparticles, HaCat and MCF-7 cells were incubated with the MMP-2-PLGA-PEI nanoparticles. The representative confocal images show that active MMP-2 was highly expressed in HaCat cells, which is an MMP-2–positive cell line, whereas active MMP-2 was barely detectable in MCF-7 cells (Figure 5A). Furthermore, the active MMP-2 signals became stronger with the increasing MMP-2-PLGA-PEI incubation time. To confirm the time-dependent active MMP-2 expression, HaCat cells were pre-incubated for 3 and 24 h. The level of active MMP-2 was approximately twice as high in 24 h precultured HaCat cells compared with 3 h precultured HaCat cells (Figure 5B). This result clearly showed the ability of the nanoparticles to differentiate the MMP-2-positive cells from the MMP-2-negative cells. Moreover, the comparison with RhoB signals allowed us to visualize dynamic MMP-2 activities from cancer cells and their surroundings. The activity of MMP-2 and the location of the nanoparticles were confirmed at the same time using dual image probes (Supplementary Figure S2). For future research based on the results of this study, the activity of MMP-2 using these nanoparticles might be visualized by in vivo experiments, which will be helpful for an early diagnosis of cancer and improvement of the molecular diagnostic technology to distinguish between malignant and nonaggressive or benign tumors.

## 4. Conclusions

This research demonstrated that MMP-2-PLGA-PEI nanoparticles were able to accurately detect MMP-2 activity of cancer cells. The use of an MMP-2-activated peptide sensor and RhoB lipid incorporated into PLGA-PEI nanoparticles allowed the detection of the active MMP-2 expression and the monitoring of the exact location of the nanoparticles. In our future research, we aim to focus on multisensor conjugated nanoparticles, including therapeutic agents.

## Supplementary Materials

The following are available online at http://www.mdpi.com/2079-4991/8/2/119/s1, Figure S1: Figure S1. The MMP-2 mRNA level in MCF-7, HaCat, and other human cancer cell lines were analyzed by RT-PCR, using GAPDH as the internal control, Figure S2: Cell culture showing the time–dependent activity of MMP-2 in HaCat cells. The cells were pre-cultured in serum-free media for 3 h and 24 h, and then incubated with MMP-2-PLGA-PEI nanoparticles, respectively, for 30 min. Scale bar: 20 µm.
