# Supplementary material for: Visualization of MMP-2 Activity Using Dual-Probe Nanoparticles to Detect Potential Metastatic Cancer Cells
Source: Nanomaterials (Basel). 2018 Feb 21;8(2):119. doi: 10.3390/nano8020119 (PMC5853750; doi:10.3390/nano8020119)
Supplement: Supplementary file 1 [file nanomaterials-08-00119-s001.pdf]

nanomaterials

Supplementary Information

Visualization of MMP-2 Activity Using Dual-Probe Nanoparticles to Detect Potential Metastatic Cancer Cells

Aeju Lee [1,2,#], Sung Hoon Kim [3,#], Hyun Lee [4], Bohee Kim [3], Yoon Suk Kim [3], Jaehong Key [4,*]

[1] International Research Organization for Advanced Science and Technology (IROAST), Kumamoto University, Japan; aeju-lee@kumamoto-u.ac.jp
[2] Magnesium Research Center, Kumamoto University, Japan; aeju-lee@kumamoto-u.ac.jp
[3] Department of Biomedical Laboratory Science, College of Health Sciences, Yonsei University, Wonju, Gangwon-do, 26493, Republic of Korea; yoonsukkim@yonsei.ac.kr
[4] Department of Biomedical Engineering, Yonsei University, Wonju, Gangwon-do, 26493, Republic of Korea; jkey@yonsei.ac.kr
* Correspondence: jkey@yonsei.ac.kr; Tel.: +82-33-760-2857
# Contributed equally to this work.

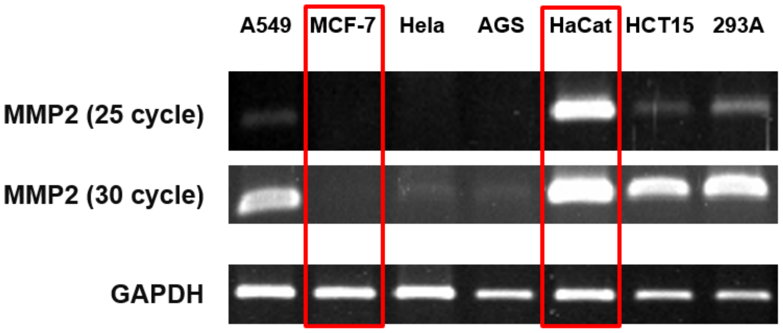

Figure S1. The MMP-2 mRNA level in MCF-7, HaCat, and other human cancer cell lines were analyzed by RT-PCR, using GAPDH as the internal control.

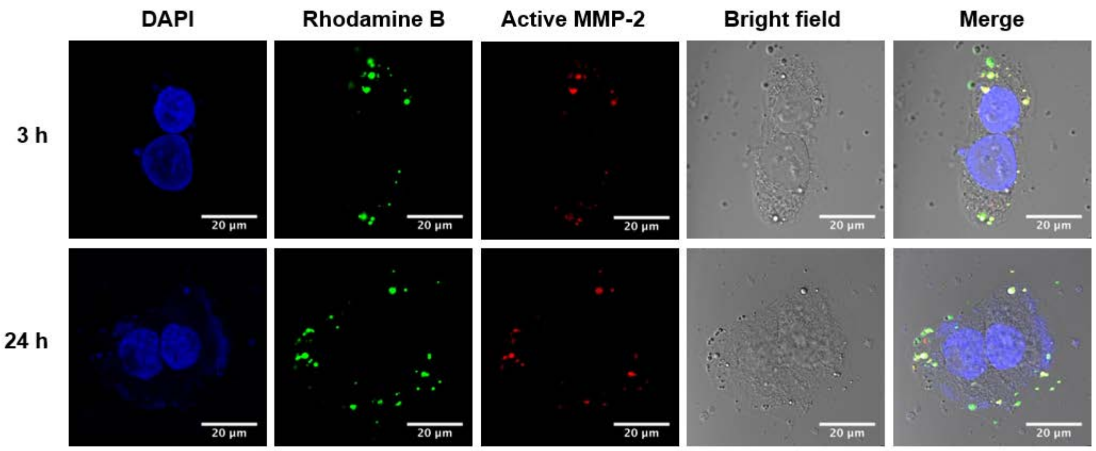

Figure S2. Cell culture showing the time–dependent activity of MMP-2 in HaCat cells. The cells were pre-cultured in serum-free media for 3 h and 24 h, and then incubated with MMP-2-PLGA-PEI nanoparticles, respectively, for 30 min. Scale bar: 20 μm.
